# Comprehensive retinal vascular measurements: a novel association with renal function in type 2 diabetic patients in China

**DOI:** 10.1038/s41598-020-70408-0

**Published:** 2020-08-13

**Authors:** Xiayu Xu, Fei Sun, Qiong Wang, Maiye Zhang, Wenxiang Ding, Aili Yang, Bin Gao

**Affiliations:** 1 The Key Laboratory of Biomedical Information Engineering of Ministry of Education, School of Life Science and Technology, Xi’an Jiaotong University, Xi’an, 710049 People’s Republic of China; 2 Bioinspired Engineering and Biomechanics Center (BEBC), Xi’an Jiaotong University, Xi’an, 710049 People’s Republic of China; 3 Department of Endocrinology and Metabolism, Xijing Hospital, Fourth Military Medical University, Xi’an, 710032 People’s Republic of China; 4 Department of Endocrinology, Tangdu Hospital, Fourth Military Medical University, Xi’an, 710038 People’s Republic of China

**Keywords:** Image processing, Endocrinology, Endocrine system and metabolic diseases, Endocrine system and metabolic diseases, Kidney diseases, Eye diseases, Retinal diseases

## Abstract

To examine the association between various retinal vascular measurements and microalbuminuria in patients with type 2 diabetes in a northwestern China study. Data from 911 patients with type 2 diabetes were analyzed. Novel retinal vascular measurements from the whole vascular tree were extracted using a validated fully automatic computer program. Retinal vascular measurements were analyzed continuously and categorically for associations with microalbuminuria using multiple logistic regressions, adjusted for related variables. In logistic regression adjusting for multiple variables, microalbuminuria was associated with smaller peripheral arteriolar caliber, larger peripheral venular caliber, larger arteriolar tortuosity, and smaller arteriolar fractal dimension (*p* = 0.028, *p* < 0.001, *p* = 0.038, *p* = 0.035, respectively). In further categorical analyses, microalbuminuria was related to smaller peripheral arteriolar caliber [T1 vs. T3: odds ratio (OR) 2.029; 95% confidence interval (CI) 1.186–3.473], larger peripheral venular caliber (T1 vs. T3: OR 0.609; 95% CI 0.362–1.024), and smaller arteriolar fractal dimension (T1 vs. T3: OR 1.659; 95% CI 1.028–2.675). Microalbuminuria in type 2 diabetes is associated with both retinal vascular caliber and geometry. These noninvasive vascular measurements serve as potential preclinical markers to identify populations at high risk of early kidney disease in the course of diabetes.

## Introduction

There are 463 million adults with diabetes mellitus worldwide in 2019, with high rates of diabetic kidney disease (DKD) reported^1^. It is shown that even early stages of DKD confer a substantial increase in the risk of cardiovascular disease, making the early identification of DKD of great importance^2^. Current biomarkers, such as albuminuria and estimated glomerular filtration rate (eGFR), are mostly functional measures and have limited predictive precision at an earlier preclinical stage^3^. Kidney biopsy samples can show early disease, but the biopsy procedure is too invasive for routine use^3^. As a result, there remains an unmet clinical need for easily accessible, non-invasive surrogate biomarkers that allow early identification of those individuals at high risk^4^.

The retina gives a unique chance to visualize and monitor human microcirculation optically and non-invasively. Moreover, retinal and renal microvasculature is reported to share similar physiological changes during early diabetes because of abnormal glucose metabolism and other processes^5,6^. In this respect, a large number of studies have been conducted in the past decades and evidences have suggested that retinal vascular changes are related to the risk of renal dysfunction in diabetes^7–10^.

In spite of the general associations that have been established, the results have been inconclusive^11^. For instance, retinal arteriolar caliber was positively associated^12–14^ or not associated^15,16^ with reduced renal function, while venular caliber was negatively associated^17,18^ or not associated^14–16^ with reduced renal function. Previous studies on retinal vasculature were usually restricted to retinal vessel from selected regions in the image. For example, caliber measurements were restricted to retinal vessels proximal to the optic disc while geometrical measurements were restricted to manually selected vascular branches^19^. However, a few recent studies have suggested that more peripheral retinal vessels may signify an even earlier change than central retinal vessels in microvascular complications of diabetes^20^. In this respect, although inspiring results were reported, to the best of our knowledge, no study has extended the measurements to include all visible vessels in the retinal image.

In this study, we examined the associations between various retinal vascular measurements and urinary albumin to creatinine ratio (ACR) in patients with type 2 diabetes. Retinal vascular measurements were extended to the most distal branches using a validated fully-automatic computer program. We hypothesized that extending the measurements to distal branches will allow a better representation of the entire vasculature and thus provide a more comprehensive understanding of the relationship between retinal vasculature and early renal function in diabetes.

## Materials and methods

### Study population and study design

The Northwest China Diabetes Study is a cross-sectional study of diabetes patient, who attended the First Affiliated Hospital (Xijing Hospital) of Fourth Military Medical University of China between January 2014 and August 2016. For this study, we included participants with type 2 diabetes, aged from 18 to 70 years old and further excluded those with prevalent cardiovascular diseases (defined as self-reported myocardial infarction, angina or stroke), diabetic ketoacidosis, diabetic hyperglycemic hyperosmolar state, septicemia and end-stage renal disease (estimated glomerular filtration rate ≤ 30 mL/(min 173 m^2^)) (n = 934). Of the 934 participants, 911 (97.5%) had gradable retinal photographs and formed the base population of this study. All data were collected with approval by the Institutional Review Board of the Air Force Medical University of China in accordance with the tenets of the Declaration of Helsinki. Informed consent was obtained from all participants.

### Measurement of early renal dysfunction

Participants provided a spot urine specimen generally immediately after arriving in the morning and urine albumin and creatinine were measured at the Clinical Chemistry Laboratory of Xijing Hospital. ACR was calculated from assayed albumin and creatinine levels. Microalbuminuria was defined as ACR > 2.5 mg/mmol in men and ACR > 3.5 mg/mmol in women^21^.

### Measurement of other variables

Digital fundus photography was performed using a 45° retinal camera (Canon CR-DGI with a 10D SLR digital camera back, Canon, Tokyo, Japan) after pupil dilation. To better visualize the distal branches, macular-centered retinal images were captured. The right eye of each participant was used and when unavailable, was replaced by the left eye.

All participants underwent a face-to-face interview at clinic regarding their past medical history and current medications. Systolic and diastolic blood pressure were measured using a digital automatic BP monitor (Orion Instruments, Inc). Body mass index (BMI) was calculated from weight (kg) divided by squared height (m^2^). Biochemical analysis of fasting venous blood samples was performed with DX-800 Automated analyzer (Beckman Kurt Inc) for HbA1c, serum creatinine, total cholesterol, triglycerides, high-density lipoprotein (HDL), and low-density lipoprotein (LDL).

### Retinal vascular measurements

**Figure 1:**
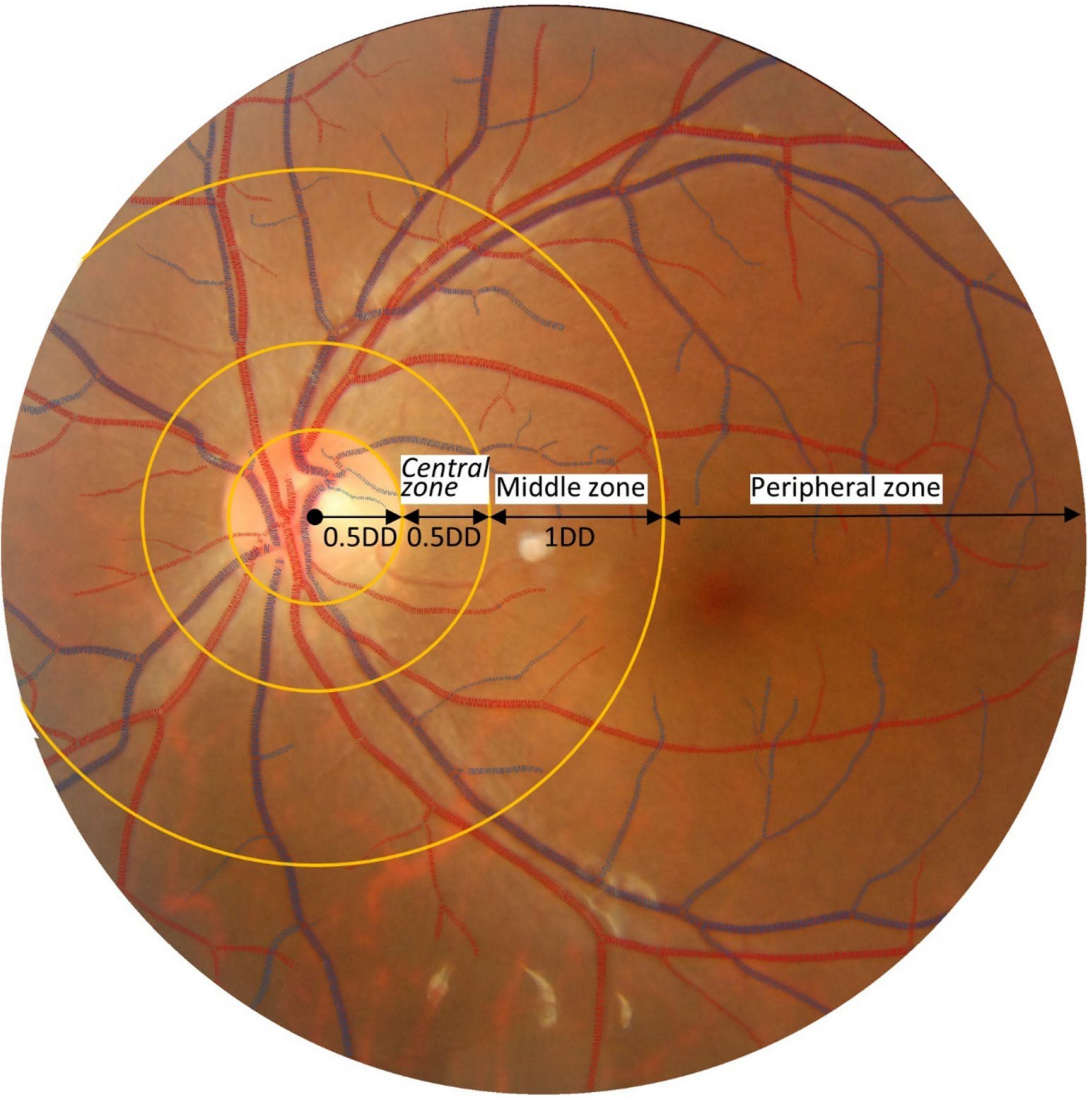
Sample image showing the vascular caliber measurements. Centered at the optic disc, blood vessels were categorized into three concentric zones: central zone (0.5–1.0 DD), middle zone (1.0–2.0 DD), and peripheral zone (> 2.0 DD). The averaged arteriolar and venular calibers were calculated in each zone and denoted as *aCtr*, *aMdl*, *aPeri*, *vCtr*, *vMdl*, and *vPeri*, respectively.

Retinal vascular measurements were performed using a fully-automated computer software (supplemental Fig. 1). The software automatically finds optic disc center, segments the whole vascular tree, distinguishes arterioles and venules, measures vessel width, and calculates tortuosity and fractal dimension. Specifically, two sets of measurements were calculated. The first set is vascular caliber measurements. Centered at the optic disc, blood vessels were categorized into three concentric zones: central zone (0.5–1.0 disc diameter, DD), middle zone (1.0–2.0 DD), and peripheral zone (> 2.0 DD) (Fig. 1). The averaged arteriolar and venular calibers were calculated in each zone and denoted as *aCtr*, *aMdl*, *aPeri*, *vCtr*, *vMdl*, and *vPeri*, respectively. The second set is vascular geometry measurements, including arteriolar and venular fractal dimension (i.e., *aD*_*f*_ and *vD*_*f*_) and tortuosity (i.e., *aTor* and *vTor*). The accuracy and reproducibility of this system have been validated extensively on public datasets^22^.

### Statistical methods

At baseline, continuous data are presented as mean ± SD and categorical data are presented as percentages. Mann–Whitney U test was used for continuous data and the χ^2^ test was used for categorical data to assess the differences between two groups. In logistic regression, vascular measurements were first analyzed as continuous variables and, if significance was found, further analyzed as categorical variables in tertiles. Two models were used to analyze the association between vascular measurements and microalbuminuria in logistic regression, with the first model adjusted for basic participant information (i.e., age and gender) and the second model further adjusted for covariates known to be associated with renal dysfunction (i.e., duration, smoking, BMI (kg/m^2^), systolic and diastolic blood pressure (mmHg), glycosylated hemoglobin (%), total cholesterol (mmol/L), HDL (mmol/L), LDL (mmol/L), and triglyceride (mmol/L)). The counterpart vessel caliber was further adjusted in the vessel caliber analysis. Findings with a *p* value < 0.05 were considered statistically significant. The 95% CI was given for estimates of ORs and considered statistically significant if they did not cross 1.0. All statistical analyses were performed using Statistical Package for the Social Sciences version 17.0 (SPSS Inc., Chicago, IL, USA).

## Results

### Baseline characteristic

Baseline characteristics are given in Table 1. Subjects without microalbuminuria were more likely to be younger, with shorter duration, lower BMI, SBP, DBP, and triglyceride. There were no significant differences in smoking, HbA1c, total cholesterol, HDL, and LDL between the two groups. Subjects with microalbuminuria showed a smaller arteriolar fractal dimension (*aD*_*f,*_) as well as larger peripheral venular caliber (*vPeri*).

**Table 1:** Demographic and clinical baseline characteristics of the study subjects.

	All	Microalbuminuria		
	(n = 911)	Absent (714)	Present (197)	*p* value
Men (%)	70.7	70.0	73.1	0.402
Age (years)	49.61 ± 10.31	49.35 ± 10.11	50.55 ± 10.98	0.049
Duration (years)	6.62 ± 5.78	6.20 ± 5.60	8.12 ± 6.19	< 0.001
Smoking (%)	47.6	47.9	46.7	0.766
Body mass index (kg/m^2^)	25.81 ± 3.44	25.63 ± 3.51	26.47 ± 3.10	0.001
SBP (mmHg)	126.44 ± 15.84	123.92 ± 14.30	135.61 ± 17.73	< 0.001
DBP (mmHg)	79.53 ± 10.20	78.58 ± 9.65	82.99 ± 11.37	< 0.001
HbA1c (%)	8.90 ± 2.25	8.85 ± 2.28	9.07 ± 2.10	0.073
Total cholesterol (mmol/L)	4.07 ± 1.01	4.07 ± 0.99	4.06 ± 1.06	0.997
Triglyceride (mmol/L)	2.03 ± 1.79	1.96 ± 1.71	2.30 ± 2.00	0.008
HDL (mmol/L)	0.96 ± 0.24	0.97 ± 0.24	0.93 ± 0.23	0.169
LDL (mmol/L)	2.39 ± 0.93	2.40 ± 0.90	2.36 ± 1.04	0.535
Geometry measurements				
* aTor*	0.46 ± 0.05	0.46 ± 0.05	0.46 ± 0.06	0.319
* vTor*	0.55 ± 0.06	0.55 ± 0.05	0.56 ± 0.06	0.163
* aD*_*f*_	1.44 ± 0.02	1.44 ± 0.02	1.43 ± 0.02	< 0.001
* vD*_*f*_	1.45 ± 0.02	1.45 ± 0.02	1.45 ± 0.02	0.111
Caliber measurements				
* aCtr* (μm)	43.25 ± 3.08	43.34 ± 2.99	42.92 ± 3.39	0.287
* aMdl* (μm)	41.50 ± 3.27	41.52 ± 3.26	41.43 ± 3.34	0.657
* aPeri* (μm)	38.33 ± 3.21	38.37 ± 3.09	38.17 ± 3.60	0.326
* vCtr* (μm)	47.53 ± 2.25	47.49 ± 2.20	47.67 ± 2.44	0.208
* vMdl* (μm)	42.20 ± 2.73	42.10 ± 2.67	42.56 ± 2.95	0.110
* vPeri* (μm)	38.78 ± 2.54	38.63 ± 2.40	39.33 ± 2.95	0.015

Continuous variables are given as mean ± SD.

MBI, body mass index; DBP, diastolic blood pressure; SBP, systolic blood pressure.

### Logistic regression

In logistic regression analyses, retinal vascular measurements were first analyzed as continuous variable (Table 2). For vascular geometry measurements, arteriolar fractal dimension was associated with microalbuminuria in Model 1 (OR 0.062; 95% CI 0.015–0.249) and Model 2 (OR 0.187; 95% CI 0.039–0.886). Arteriolar tortuosity was associated with microalbuminuria after adjustment in Model 2 (OR 32.733; 95% CI 1.217–880.629). Venular tortuosity was also associated with microalbuminuria after adjustment in Model 1 (OR 18.860, 95% CI 1.249–284.667), but the association was attenuated and no longer significant after adjusting for more risk factors in Model 2.

**Table 2:** Adjusted odds ratios of microalbuminuria in relation to retinal vascular measurements as continuous variables.

	Microalbuminuria			
	Model 1*	*p* value	Model 2**	*p* value
	OR (95% CI)		OR (95% CI)	
Geometry measurements				
* aTor*	11.703 (0.671,204.053)	0.092	32.733 (1.217,880.629)	0.038
* vTor*	18.860 (1.249,284.667)	0.034	7.837 (0.358,171.715)	0.191
* aD*_*f*_	0.062 (0.015,0.249)	< 0.001	0.187 (0.039,0.886)	0.035
* vD*_*f*_	0.420 (0.109,1.620)	0.208	0.556 (0.114,2.700)	0.466
Caliber measurements				
* aCtr* (μm)^†^	0.935 (0.885,0.988)	0.016	0.954 (0.895,1.016)	0.139
* aMdl* (μm)^†^	0.945 (0.895,0.999)	0.047	0.960 (0.903,1.020)	0.185
* aPeri* (μm)^†^	0.907 (0.852,0.965)	0.002	0.924 (0.861,0.992)	0.028
* vCtr* (μm)^‡^	1.063 (0.983,1.151)	0.127	1.057 (0.967,1.155)	0.225
* vMdl* (μm)^‡^	1.113 (1.034,1.199)	0.004	1.087 (0.972,1.149)	0.194
* vPeri* (μm)^‡^	1.183 (1.096,1.278)	< 0.001	1.180 (1.080,1.290)	< 0.001

*Model 1 adjusted for age and sex.

**Model 2 adjusted for age, sex, duration, smoking, BMI, HbA1c, SBP, DBP, total cholesterol, triglyceride, high-density and low-density lipoprotein.

^†^Arteriolar caliber measurements were further adjusted for *vCtr*, *vMdl*, and *vPeri*.

^‡^Venular caliber measurements were further adjusted for *aCtr*, *aMdl*, and *aPeri*.

For vascular caliber measurements, peripheral arteriolar caliber was associated with microalbuminuria in both Model 1 (OR 0.907; 95% CI 0.852–0.965) and Model 2 (OR 0.924; 95% CI 0.861–0.992). Peripheral venular caliber was also associated with microalbuminuria in both Model 1 (OR 1.183; 95% CI 1.096–1.278) and Model 2 (OR 1.180; 95% CI 1.080–1.290). Arteriolar calibers in the central zone (OR 0.935; 95% CI 0.885–0.988) and middle zone (OR 0.945; 95% CI 0.895–0.999), as well as venular caliber in the middle zone (OR 1.113; 95% CI 1.034–1.199), were also associated with microalbuminuria in Model 1, but the association was attenuated and no longer significant after adjusting for more risk factors in Model 2.

Retinal vascular measurements showed significant association with microalbuminuria in Model 2 were further analyzed as categorical variables in tertile (Table 3). Participants at the lowest *aD*_*f*_ tertile showed 96.1% and 65.9% increased odds of microalbuminuria compared with those at the highest aDf tertile, after adjustment in Model 1 and Model 2 (OR 1.961; 95% CI 1.297–2.964 and OR 1.659; 95% CI 1.028–2.675, respectively). No associations were found between microalbuminuria and aTor.

**Table 3:** Adjusted odds ratio of microalbuminuria in relation to selected retinal vascular measurements in tertile.

	Model 1		Model 2	
	OR (95% CI)	*p* value	OR (95% CI)	*p* value
*aTor*				
T1 (0.35–0.42)	0.727 (0.489,1.081)	0.115	0.724 (0.462,1.135)	0.160
T2 (0.43–0.46)	0.817 (0.556,1.200)	0.303	0.781(0.501,1.216)	0.274
T3 (0.47–0.71)	1.00		1.00	
*aD*_*f*_				
T1 (1.32–1.42)	1.961 (1.297,2.964)	0.001	1.659 (1.028,2.675)	0.038
T2 (1.43–1.45)	1.248 (0.823,1.893)	0.297	1.225 (0.765,1.961)	0.398
T3 (1.46–1.51)	1.00		1.00	
*aPeri* (μm)^†^				
T1 (36.79–45.41)	2.200 (1.373,3.525)	0.001	2.029 (1.186,3.473)	0.010
T2 (45.42–49.18)	1.240 (0.808,1.901)	0.325	1.406 (0.861,2.297)	0.173
T3 (49.19–61.68)	1.00		1.00	
*vPeri* (μm)^‡^				
T1 (39.37–46.55)	0.651 (0.417,1.015)	0.058	0.609 (0.362,1.024)	0.062
T2 (46.56–49.15)	0.420 (0.275,0.641)	< 0.001	0.426 (0.264,0.687)	< 0.001
T3 (49.16–59.01)	1.00		1.00	

*Model 1 adjusted for age and sex;

**Model 2 adjusted for age, sex, duration, smoking, BMI, HbA1c, SBP, DBP, total cholesterol, triglyceride, high-density and low-density lipoprotein;

^†^Arteriolar caliber measurements were further adjusted for *vCtr*, *vMdl*, and *vPeri*.

^‡^Venular caliber measurements were further adjusted for *aCtr*, *aMdl*, and *aPeri*.

Participants at the lowest aPeri tertile was associated with 120.0% and 102.9% increased odds of microalbuminuria compared with those at the highest aPeri tertile, after adjustment in Model 1 and Model 2 (OR 2.200; 95% CI 1.373–3.525 and OR 2.029; 95% CI 1.186–3.473, respectively). Participants at the middle vPeri tertile showed 58.0% and 57.4% decreased odds compared with those at the highest vPeri tertile, after adjustment in Model 1 and Model 2 (OR 0.420; 95% CI 0.275–0.641 and OR 0.426; 95% CI 0.264–0.687, respectively).

## Discussion

In this study, we extended the vascular measurements to the entire vascular tree and explored the associations between microalbuminuria and retinal vascular measurements in patients with type 2 diabetes. Our findings suggested that microalbuminuria is associated with retinal vasculatures, both in terms of caliber and geometry. Our study adds significant new knowledge in this study field. First, we demonstrated that peripheral vascular calibers, rather than central vascular calibers, were strongly associated with microalbuminuria. Second, we demonstrated that arteriolar geometries, rather than venular geometries, were strongly associated with microalbuminuria.

Previous studies have been focusing on central retinal vessels proximal to optic disc^19^. As discussed in a recent study, reducing the measurement area would result in an enormous data reduction that may miss vessel changes occurring elsewhere in the retina^20^. By applying a fully automatic image processing program, we extended the measurements to the entire vascular trees, especially to the peripheral zone. Our findings showed that microalbuminuria was associated with peripheral arteriolar and venular calibers, rather than vascular calibers in the central or middle zones. It is known that smaller peripheral retinal vessels host pericytes and endothelial cells, both playing a major role in the pathogenesis of microvascular complications^23^. Apoptosis of pericytes is noted as one of the earliest vascular changes in hyperglycaemia and eventually leads to vascular nonperfusion^24^. We hypothesize that these early changes in pericytes lead to observable vascular caliber changes prior to the onset of clinical complications. In further analyses as categorical variables, the smallest *aPeri* tertile was associated with increased odds of microalbuminuria (T1 vs. T3: OR 2.029; 95% CI 1.186–3.473). These changes in peripheral vascular caliber may serve as useful biomarkers in identifying early renal changes.

Fractal dimension, a geometrical measurement indicating the complexity of branching patterns, has been associated with the incidence of diabetic nephropathy in cross-sectional and longitudinal studies^25,26^. However, to the best of our knowledge, no previous studies have separately analyzed the arteriolar and venular trees. With the automatic program, we analyzed the arteriolar and venular trees separately for the first time. We demonstrated that decreased arteriolar fractal dimension was significantly associated with reduced renal function, while the venous fractal dimension was not associated with renal function. Decreased vascular fractal dimension indicates vessel rarefaction^27^, which was speculated to be the result of vasoconstriction induced by endothelial dysfunction^28^. Although the underlying differences between arterioles and venules were not clear, it is reported that the retinal blood flow is decreased in the arterioles, but increased or unchanged in the venules in early diabetes^29–31^, which might lead to different physiological changes and results in a faster damage in arterioles.

Increased retinal arteriolar tortuosity has been associated with early kidney dysfunction in type 1 diabetes subjects^32^. But the results were based on selected arteriolar branches and could not represent the overall retinal vascular tortuosity. We extended the measurement to the entire arteriolar and venular trees for the first time. Our results supported that microalbuminuria was associated with increased arteriolar tortuosity in continuous analyses, though no association was found in further categorical analyses. It is reported that increased tortuosity is linked to impaired vessel autoregulation^33^, which is the result of disturbed blood flow and endothelial dysfunction in the course of diabetes^34^.

There are several limitations of our study. First, given the cross-sectional nature of this study, these associations do not allow a risk stratification. To gain better insights into the pathophysiological mechanism of the earlier stages of the disease and to evaluate the prognostic value of the image biomarkers, a longitudinal follow-up is desired in the future. Second, the current vascular caliber measurement simply averaged all vascular calibers within each zone and did not take the different branching levels into consideration. In this respect, caliber measurement that can represent different vascular branching levels will probably provide more meaningful information and is more desired.

In conclusion, this study evaluated the associations between various retinal vascular measurements and renal function in a northwestern China study with type 2 diabetes mellitus. The present study extended the conventional analysis of proximal branches of central retinal vessels to the entire vascular tree. We found that peripheral vascular calibers as well as arteriolar geometries were strongly associated with microalbuminuria. These measurements may assist in identifying individuals at high risk of complications early in the course of diabetes.

## Supplementary information

Supplementary file

## Data Availability

The datasets used and/or analyzed during the current study are available from the corresponding author on reasonable request.
