# Supplementary material for: Comprehensive retinal vascular measurements: a novel association with renal function in type 2 diabetic patients in China
Source: Sci Rep. 2020 Aug 13;10:13737. doi: 10.1038/s41598-020-70408-0 (PMC7426409; doi:10.1038/s41598-020-70408-0)
Supplement: Supplementary file 1 — Supplementary file [file 41598_2020_70408_MOESM1_ESM.docx]

**Comprehensive retinal vascular measurements: a novel association with renal function in type 2 diabetic patients in China**

Xiayu Xu^1,2*^, Fei Sun^3*^, Qiong Wang^3^, Maiye Zhang^3^, Wenxiang Ding^1,2^, Aili Yang^4^, Bin Gao^4#^

*^1^ The Key Laboratory of Biomedical Information Engineering of Ministry of Education, School of Life Science and Technology, Xi’an Jiaotong University, Xi’an 710049, P.R. China*

*^2^ Bioinspired Engineering and Biomechanics Center (BEBC), Xi’an Jiaotong University, Xi’an 710049, P.R. China*

*^3^ Department of Endocrinology and Metabolism, Xijing Hospital, Fourth Military Medical University, Xi’an 710032, P.R. China*

*^4^ Department of Endocrinology, Tangdu Hospital，Fourth Military Medical University, Xi’an 710038, P.R. China*

** The authors contributed equally*

*^#^ Corresponding author: bingao@fmmu.edu.cn*

1. **Algorithm Details**

Retinal vascular measurements were performed using a fully-automated computer program developed by our group. **Supplemental Figure 1** illustrates the image processing and measurement quantification processes. The arteriolar and venular trees were segmented using a validated deep learning algorithm, followed by an accurate vessel caliber measurement using a graph-theoretic method.

**1.1 Arteriovenous segmentation**

We employ the fully convolutional network (FCN) architecture to simultaneously segment both arteriole trees and venule trees in the retinal image [[1](#_ENREF_1)]. All color channels (i.e., red, green, and blue) are fused to allow the usage of full color information. In a pilot study, it is noticed that fine vessels are easily missed and that arterioles are more easily to be misclassified as venules. We thus designed the loss function to enhance the detection ability of the network on fine vessels as well as arteriole vessels. Image normalization by histogram matching is applied to eliminate the background differences. The original FCN architecture can be recognized as two parts, the descending part and the ascending part, with a total number of 23 convolutional layers. However, in the original network, the feature map after each convolution loses its boundary pixels, resulting in different input and output image size. We apply same padding in convolution to avoid inconsistent image sizes. The activation function is rectified linear unit (ReLU).

**1.2 Vessel caliber determination**

In order to detect both boundaries simultaneously, we build the graph as a two-slice 3-D graph [[2](#_ENREF_2)]. A smoothness constraint between the two slices is applied. Thus, a simultaneous two-D boundary segmentation is transformed into a two-slice 3-D surface segmentation problem. This problem is then further converted into the problem of computing a minimum closed set in a node-weighted graph. A sequential thinning approach is then applied to the binary vessel segmentation to find the vessel centerlines. From this vessel centerline image, the bifurcation points and crossing points are excluded. For each labeled vessel segment, the growing direction for every centerline pixel is calculated. Using the centerline pixels as the base nodes, profiles on the positive direction of the normals are built as one slice and profiles on the negative direction are built as another slice. Along each normal profile Col(x,y), every node V(x,y,z)(z>0) has a directed arc to the node V(x,y,z-1). Along the x-direction, a directed arc is constructed from V(x,y,z)∈Col(x,y) to V(x+1,y,max(0,z-∆x))∈Col(x+1,y). Similarly, arcs from V(x,y,z)∈Col(x,y) to V(x-1,y,max(0,z-∆x))∈Col(x-1,y,z) is constructed. ∆x is the maximum difference allowed between two adjacent normal profiles within one boundary. Along the y-direction, meaning between the two slices, arcs from V(x,y,z)∈Col(x,y) to V(x,y+1,max(0,∆y))∈Col(x,y+1,z) and arcs from V(x,y,z)∈Col(x,y) to V(x,y-1, max(0,z-∆y))∈Col(x,y-1,z) is constructed. ∆y is the maximum difference allowed between two corresponding normal profiles between the two boundaries.

An optimal surface is defined as the surface with the minimum cost among all feasible surfaces defined in the 3-D volume. The cost image is generated from the orientation sensitive 1-D first-order derivative of Gaussian of the green channel. After the node-weighted directed graph is constructed, the optimal surface is determined. To determine the vessel width, the coordinate difference between the two corresponding nodes on the optimal surface from the two slices is calculated.

1. **Vascular measurements**

From the established arteriolar and venular trees, two sets of measurements were extracted, including vascular caliber and vascular geometry. The first set is vascular calibers. Centered at the optic disc, blood vessels were categorized into three concentric zones: center zone (0.5-1.5 disc diameter, DD), middle zone (1.5-2.5 DD), and peripheral zone (>2.5 DD). The averaged arteriolar and venular calibers were calculated in each zone and denoted as *aCtr*, *aMdl*, *aPeri*, *vCtr*, *vMdl*, and *vPeri*, respectively.

The second set is vascular geometrical measurements including separated arteriolar and venular fractal dimension and separated arteriolar and venular tortuosity. Fractal dimension was implemented by applying a box-counting method [[3](#_ENREF_3)]. The arteriolar and venular fractal dimensions were calculated separately as *aD_f_* and *vD_f_*. Vessel tortuosity was calculated as the averaged integrated curvature of all vessel segments [[4](#_ENREF_4)] and the arteriolar and venular tortuosity were calculated separately as *aTor* and *vTor*.

1. **Study Description**

The Northwest China Diabetes Study is a cross-sectional study between January 2011 and August 2016 with a total number of 2,397 diabetes patients, who attended the First Affiliated Hospital (Xijing Hospital) of Air Force Medical University of China. The ACR test was launched at the end of 2014. For this study, we included participants with type 2 diabetes, aged from 18 to 70 years old and further excluded those with prevalent cardiovascular diseases (defined as self-reported myocardial infarction, angina or stroke), diabetic ketoacidosis, diabetic hyperglycemic hyperosmolar state, septicemia and end-stage renal disease (estimated glomerular filtration rate≤30ml/ (min·173m2)) (n=934). Of the 934 participants, 911 (97.5%) had gradable retinal photographs and formed the base population of this study. We tested the baseline values between the participants included and excluded in this study as given below. No differences were found between the basic factors, such as sex, age, BMI, and HbA1c and the 911 participants with ACR tests were analyzed in this study.

**Table 1. Baseline characteristics of subjects with and without ACR tests.**

|  |  | **Study**  **Participants**  **(911)** | **Missing ACR**  **(1428)** | ***p* value** |
| --- | --- | --- | --- | --- |
| Men（%） |  | 70.7 | 73.1 | 0.485 |
| Age (years) |  | 49.35±10.11 | 50.15±10.98 | 0.129 |
| Duration (years) |  | **6.62±5.78** | **5.65±5.23** | **<0.001** |
| Body mass index (kg/m^2^) |  | 25.81±3.44 | 24.90±3.52 | 0.310 |
| SBP (mmHg) |  | 126.44±15.84 | 127.01±16.09 | 0.056 |
| DBP (mmHg) |  | **79.53±10.20** | **82.99±11.37** | **0.009** |
| HbA1c (%) |  | 8.90±2.25 | 9.09±2.20 | 0.079 |

Supplemental Figure 1. Flowchart of the image processing and RIMs measurement processes.


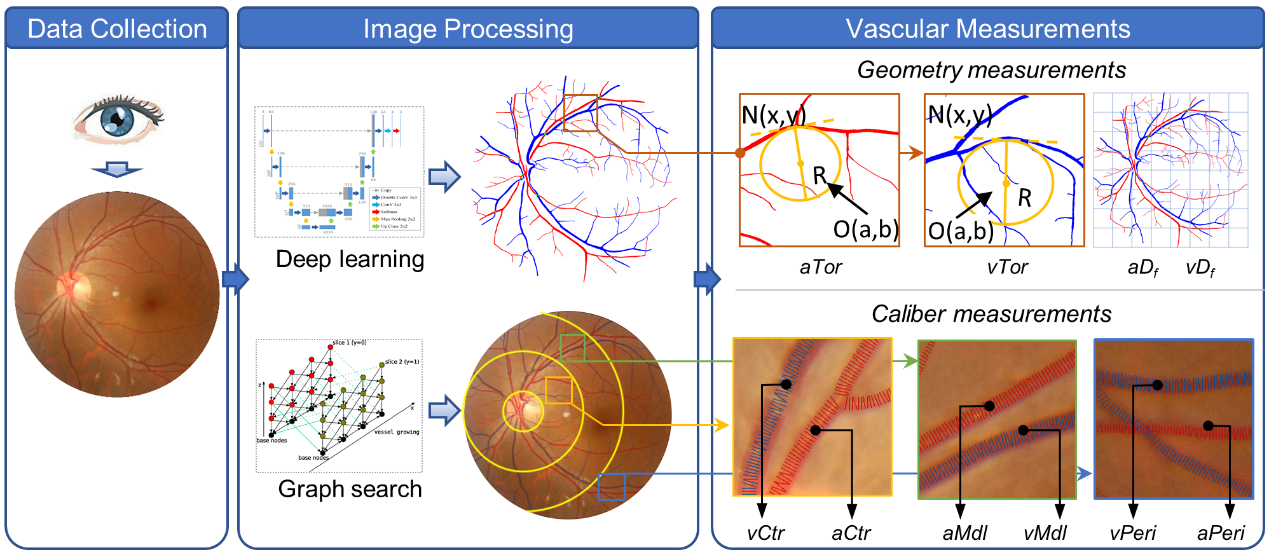


1. Xu, X., et al., *Simultaneous arteriole and venule segmentation with domain-specific loss function on a new public database.* Biomedical optics express, 2018. **9**(7): p. 3153-3166.

2. Xu, X., et al., *Vessel Boundary Delineation On Fundus Images Using Graph-Based Approach.* Medical Imaging, IEEE Transactions on, 2011. **30**(6): p. 1184-1191.

3. Stosic, T. and B.D. Stosic, *Multifractal analysis of human retinal vessels.* IEEE Transactions on Medical Imaging, 2006. **25**(8): p. 1101-1107.

4. Hart, W.E., et al., *Measurement and classification of retinal vascular tortuosity.* International Journal of Medical Informatics, 1999. **53**(2-3): p. 239-252.
